# A clinical predictive model for risk stratification of patients with severe acute lower gastrointestinal bleeding

**DOI:** 10.1186/s13017-021-00402-y

**Published:** 2021-11-22

**Authors:** Manraj Singh, Jayne Chiang, Andre Seah, Nan Liu, Ronnie Mathew, Sachin Mathur

**Affiliations:** 1 Department of General Surgery, Singapore General Hospital, 20 College Rd, Singapore, 169856 Singapore; 2 Health Services Research Centre, Singapore Health Services, Singapore, Singapore; 3 Department of Colorectal Surgery, Singapore General Hospital, Singapore, Singapore; 4 Department of Trauma and Acute Care Surgery, Singapore General Hospital, Singapore, Singapore

**Keywords:** Lower gastrointestinal bleeding, Predictive model, Per-rectal bleed, Haematochezia, Haemorrhagic, Shock

## Abstract

**Background:**

Lower gastrointestinal bleeding (LGIB) is a common presentation of surgical admissions, imposing a significant burden on healthcare costs and resources. There is a paucity of standardised clinical predictive tools available for the initial assessment and risk stratification of patients with LGIB. We propose a simple clinical scoring model to prognosticate patients at risk of severe LGIB and an algorithm to guide management of such patients.

**Methods:**

A retrospective cohort study was conducted, identifying consecutive patients admitted to our institution for LGIB over a 1-year period. Baseline demographics, clinical parameters at initial presentation and treatment interventions were recorded. Multivariate logistic regression was performed to identify factors predictive of severe LGIB. A clinical management algorithm was developed to discriminate between patients requiring admission, and to guide endoscopic, angiographic and/or surgical intervention.

**Results:**

226/649 (34.8%) patients had severe LGIB. Six variables were entered into a clinical predictive model for risk stratification of LGIB: Tachycardia (HR ≥ 100), hypotension (SBP < 90 mmHg), anaemia (Hb < 9 g/dL), metabolic acidosis, use of antiplatelet/anticoagulants, and active per-rectal bleeding. The optimum cut-off score of ≥ 1 had a sensitivity of 91.9%, specificity of 39.8%, and positive and negative predictive Values of 45% and 90.2%, respectively, for predicting severe LGIB. The area under curve (AUC) was 0.77.

**Conclusion:**

Early diagnosis and management of severe LGIB remains a challenge for the acute care surgeon. The predictive model described comprises objective clinical parameters routinely obtained at initial triage to guide risk stratification, disposition and inpatient management of patients.

## Background

Lower gastrointestinal bleeding (LGIB), defined as bleeding distal to the ligament of Treitz, remains a common presenting symptom for emergency general surgical patients. The annual incidence (20–30 cases per 100,000 adults) rises 200-fold between the 3rd and 9th decade of life [1, 2]. Amongst the elderly, the morbidity secondary to LGIB is exacerbated due to the interplay of multiple comorbidities, use of antiplatelet and/or anticoagulants and poor functional reserves [3].

The complexity in management of patients with LGIB relates to the wide spectrum of aetiologies, spanning benign and malignant disease, affecting the small and large intestine as well as anal canal. The presentation varies widely between stable haemorrhoidal bleeding requiring outpatient management to exsanguinating colonic bleeding that may require a colectomy. For these presentations and everything in between, a well-developed diagnostic, investigative and therapeutic strategy is required to resuscitate, localise and then treat the underlying pathology. As there is no ‘one size fits all’, LGIB management remains difficult to protocolise.

Timely recognition of severe LGIB is crucial in implementing effective management pathways. However, few clinical predictive tools for prognostication of LGIB exist in the literature. Furthermore, none have been validated in Asian populations [4]. In this study, we aim to identify predictors of severe LGIB and develop a predictive model. Furthermore, we aim to develop an algorithm for the management of patients with LGIB.

## Materials and methods

A retrospective cohort study was conducted of adult (≥ 21 years old) patients admitted via the Emergency Department (ED) to our institution over a 12-month period from July 2016 to June 2017. Patients with an ICD coding of “lower gastrointestinal bleeding” (LGIB) or “per-rectal bleeding” from the ED database and inpatient discharge summaries were identified.

LGIB was defined as gastrointestinal bleeding originating distal to the ligament of Treitz confirmed via digital per-rectal examination, proctoscopy and/or endoscopy. Our definition of severe LGIB was modified from Strate’s: Presence of bleeding necessitating 2 or more units of packed red blood cell transfusion within the first 24-h of admission, re-bleeding after 24-h of clinical stability and/or the need for additional transfusion beyond 24-h [5]. Those with UGIB (upper gastrointestinal bleeding), as defined by the presence of hematemesis, melena and with endoscopic confirmation of a bleeding source proximal to the ligament of Treitz were excluded. The primary outcome was to elucidate clinical parameters and factors predictive of severe LGIB. The secondary outcome was to construct a clinical predictive model to risk stratify these patients. The study protocol was approved by the local Institutional Review Board.

### Statistical analysis

Patient demographics, clinical parameters and biochemistry on admission were presented as dichotomised variables. Categorical variables were analysed with the Pearson *X*^2^ or Fisher exact test, while continuous variables were analysed with a paired *T*-test or Mann–Whitney *U*. Severe and non-severe LGIB were used to stratify the aetiology of bleeding, therapeutic intervention as well as severity outcome measures such as mortality and ICU admission.

Univariate predictors of severe LGIB were determined and those that were statistically significant entered into a multivariate logistic regression model using backward selection. Odds ratios (OR) were generated for the effect of individual variables with 95% confidence intervals. Factors significant on multivariable analysis were incorporated into a 6-point clinical predictive model. The sensitivity, specificity, positive (PPV) and negative predictive values (PPV) were calculated based on the cumulative increase in score of the model. A Receiver Operating Statistics (ROC) curve was plotted and the area under curve (AUC) calculated to assess the performance of the model in predicting severe LGIB.

*P* values less than 0.05 were considered statistically significant. All statistical analysis was performed using SPSS Statistics for Windows v25.0 (Armonk, NY: IBM Corp).

## Results

There were 649 patients admitted with acute LGIB during the study period, of which 226 (34.8%) were designated severe and 423 (65.2%) non-severe. The demographics and clinical parameters are described in Table 1. Most patients (*n* = 469/649, 72.3%) were above 60 years of age [mean 67(± SD15)]. The M:F ratio was 54:46, respectively, and the majority were of Chinese ethnicity (*n* = 576/649, 88.8%). Almost two-thirds of patients had a Charlson Comorbidity (CCM) [6] score of 2 or more, which had higher incidence in those with severe LGIB (71.2% vs. 64.3%, *P* = 0.07). The use of antiplatelet or anticoagulant medications was higher in the severe LGIB cohort (38.1% vs. 29.1%, *P* = 0.02).

**Table 1:** Demographics of study population

Variable	Total cohort *n* = 649 (%)	Severe bleed *n* = 226 (%)	Non-severe bleed *n* = 423 (%)	*P* value
Mean age, y (± SD)	67.3 (15.2)	68.3 (15.0)	66.7 (15.3)	0.65
< 60	180 (27.7)	59 (26.1)	121 (28.6)	
≥ 60	469 (72.3)	167 (73.9)	302 (71.4)	0.50
Gender				
Male	351 (54.1)	121 (53.5)	230 (54.4)	0.84
Female	298 (45.9)	105 (46.5)	193 (45.6)	
Race				
Chinese	576 (88.8)	203 (89.8)	373 (88.2)	0.053
Malay	40 (6.2)	18 (8.0)	22 (5.2)	
Indian	16 (2.5)	3 (1.3)	13 (3.1)	
Others	17 (2.6)	2 (0.9)	15 (3.5)	
CCM score				
≤ 2	216 (33.3)	65 (28.8)	151 (35.7)	0.074
> 2	433 (66.7)	161 (71.2)	272 (64.3)	
CKD	67 (10.3)	33 (14.6)	34 (8.0)	0.009
Recent NSAID use	4 (0.6)	0 (0)	4 (0.9)	0.14
Antiplatelet/coagulant use	209 (32.2)	86 (38.1)	123 (29.1)	0.02
Median duration of bleeding, days (IQR)	1 (1–4)	1 (1–3)	2 (1–4)	0.65

*CCM* Charlson comorbidity, *CKD* chronic kidney disease, *NSAID* non-steroidal anti-inflammatory drugs, *IQR* inter-quartile range

At initial presentation in ED, patients in the severe LGIB cohort were more likely to have active per-rectal bleeding (43.8% vs. 30.3%, *P* = 0.001, Table 2), tachycardia (HR ≥ 100, 22.6% vs. 9.5%, *P* < 0.001), hypotension (SBP < 90 mmHg, 6.2% vs. 0.5%, *P* < 0.001) and anaemia (Hb < 9 g/dL, 46.9% vs. 5.0%, *P* < 0.001). Acute Kidney Injury (AKI) was seen in a third of patients, with 45.1% in the severe cohort versus 27.0% in the non-severe group (*P* < 0.001). Metabolic acidosis, as reflected by a low serum bicarbonate, was seen more frequently in the severe LGIB cohort (12.8% vs. 3.8%, *P* < 0.001). Two patients (0.3%) presented with cardiovascular collapse secondary to ongoing rapid haemorrhage.

**Table 2:** Mean clinical parameters on admission

Variable	Total cohort *n* = 649 (%)	Severe bleed *n* = 226 (%)	Non-severe bleed *n* = 423 (%)	*P* value
HR (SD)	83 (16)	86 (17)	81 (15)	0.009
≥ 100	91 (14.0)	51 (22.6)	40 (9.5)	
< 100	558 (86.0)	175 (77.4)	383 (90.5)	< 0.001
SBP, mmHg (SD)	134.8 (25.3)	126 (27)	140 (23)	0.002
< 90	16 (2.5)	14 (6.2)	2 (0.5)	
≥ 90	633 (97.5)	212 (93.8)	421 (99.5)	< 0.001
DBP, mmHg (SD)	72 (13)	66 (13)	75 (12)	0.001
MAP, mmHg (SD)	93 (16)	86 (16)	96 (14)	0.48
< 65	14 (2.2)	11 (4.9)	3 (0.7)	
≥ 65	632 (97.4)	212 (93.8)	420 (99.3)	0.001
Hb, g/dL (SD)	11.3 (2.8)	9.1 (2.6)	12.5 (2.1)	< 0.001
< 9	127 (19.6)	106 (46.9)	21 (5.0)	
≥ 9	521 (80.3)	120 (53.1)	401 (94.8)	< 0.001
Hct, % (SD)	34.4 (7.7)	28.5 (7.4)	37.6 (5.8)	< 0.001
> 35	344 (53.0)	46 (20.4)	298 (70.4)	
≤ 35	304 (46.8)	180 (79.6)	124 (29.3)	< 0.001
AKI	216 (33.3)	102 (45.1)	114 (27.0)	< 0.001
Coagulopathy, INR ≥ 1.5	27 (4.2)	14 (6.2)	13 (3.1)	0.08
HCO_3_, mEq/L (SD)	23.9 (3.0)	22.8 (3.3)	24.5 (2.6)	0.003
≤ 19	45 (6.9)	29 (12.8)	16 (3.8)	
> 19	594 (91.5)	193 (85.4)	401 (94.8)	< 0.001
Active PR bleed	227 (35.0)	99 (43.8)	128 (30.3)	0.001
Cardiovascular collapse	2 (0.3)	2 (0.9)	0 (0)	0.12

*HR* heart rate, *SBP* systolic blood pressure, *DBP* diastolic blood pressure, *MAP* mean arterial pressure, *Hb* haemoglobin, *Hct* haematocrit, *AKI* acute kidney injury, *INR* international normalised ratio, *HCO*_*3*_ bicarbonate (acidosis)

The distribution of aetiologies for LGIB is described in Table 3. The majority of patients had bleeding secondary to haemorrhoids (36.4%), diverticular disease (32.5%) and colorectal cancer (15.1%). Less frequent causes included colitis and proctitis (9.2%), post-polypectomy or haemorrhoidectomy bleeding (1.4%), solitary rectal ulcers (SRUS, 1.7%) and small bowel bleed (0.3%). There was a higher incidence of diverticular bleeding in the severe LGIB group (46.9% vs. 24.8%). The majority of non-severe LGIB were due to haemorrhoids (42.6%). Sixteen (2.4%) patients had inconclusive investigations, or declined workup due to age or financial concerns. We postulate that a number of these were AVMs (Arteriovenous Malformation).

**Table 3:** Etiology of LGIB

	Total cohort *n* = 649 (%)	Severe bleed *n* = 226 (%)	Non-severe bleed *n* = 423 (%)
Haemorrhoids	236 (36.4)	56 (24.8)	180 (42.6)
Diverticular disease	211 (32.5)	106 (46.9)	105 (24.8)
Colorectal malignancy	98 (15.1)	32 (7.6)	66 (15.6)
Colitis	39 (6.0)	7 (3.1)	32 (7.6)
Radiation proctitis	21 (3.2)	6 (2.7)	15 (3.5)
SRUS	11 (1.7)	5 (2.2)	6 (1.4)
Postoperative bleeding^a^	9 (1.4)	3 (1.3)	6 (1.4)
Perianal disease^b^	4 (0.6)	1 (0.4)	3 (0.7)
Rectal prolapse	1 (0.1)	0 (0)	1 (0.2)
Abernathy lesion	1 (0.1)	1 (0.4)	0 (0)
Small bowel bleed	2 (0.3)	2 (0.9)	0 (0)
Unknown (includes AVM)	16 (2.4)	7 (3.1)	9 (2.1)

*SRUS* solitary rectal ulcer syndrome, *AVM* arteriovenous malformation

^a^Post-op bleeding (post-polypectomy, haemorrhoidectomy); ^b^Perianal fissure, hematoma or fistula

Table 4 outlines the differences in therapeutic intervention and clinical outcomes between severe and non-severe LGIB patients. More than a third of patients required packed cells transfusion (*n* = 212/649, 36.7%), with 89.1% (*n* = 212/238) receiving their first transfusion within 24 h-h, and 65.9% (*n* = 149/226) of those with severe LGIB receiving 2 or more units. Overall, 362 patients (55.8%) underwent endoscopic evaluation during the admission of which 123 (19%) were performed within 24-h (24H). More patients in the severe LGIB group underwent endoscopic evaluation (65.5% vs. 50.6%, *P* < 0.001). There was no significant difference in the incidence of early endoscopy within 24-h between both strata. Eleven patients (1.7%) underwent angio-embolisation, all of whom were from the severe LGIB cohort—2 received it within 24-h of admission.

**Table 4:** Severity outcome measures and therapeutic interventions

Variable	Total cohort (*n* = 649)	Severe bleed (*n* = 226)	Non-severe bleed (*n* = 423)	*P* value
Rebleeding during admission	106 (16.3)	106 (46.9)	0 (0)	< 0.001
Required blood transfusion	238 (36.7)	204 (90.3)	34 (8.0)	< 0.001
Blood transfusion within 24H	212 (32.7)	178 (78.8)	34 (8.0)	< 0.001
≥ 2 PCT	149 (23.0)	149 (65.9)	0 (0)	< 0.001
Median PCT (IQR)	0 (0–2)	2 (2–4)	0 (0)	< 0.001
Endoscopy	362 (55.8)	148 (65.5)	214 (50.6)	< 0.001
OGD	190 (29.3)	107 (47.3)	83 (19.6)	< 0.001
Colonoscopy	314 (48.4)	121 (53.5)	193 (45.6)	0.06
Sigmoidoscopy	42 (6.5)	21 (9.3)	21 (5.0)	0.033
Endoscopy < 24H	123 (19.0)	42 (18.6)	81 (19.1)	0.86
Surgery	48 (7.4)	23 (10.2)	25 (5.9)	0.048
Surgery < 24H	12 (1.8)	3 (1.3)	9 (2.1)	0.56
Angioembolisation	11 (1.7)	11 (4.9)	0 (0)	< 0.001
Angioembolisation < 24H	2 (0.3)	2 (0.9)	0 (0)	0.12
ICU stay < 24H	5 (0.8)	5 (2.2)	0 (0)	0.005
Median length of stay (IQR)	3 (2–5)	5 (3–7)	3 (2–4)	< 0.001
72H mortality	1 (0.2)	1 (0.4)	0 (0)	0.35
30 day mortality	3 (0.5)	3 (1.3)	0 (0)	0.042

Values in parentheses are percentages

*PCT* packed red blood cell transfusion, in units

Five patients (0.8%) required admission to the Intensive Care Unit (ICU). The 72-h mortality rate was 0.2% (1 patient) and 30-day mortality was 0.5% (3 patients). Eleven patients (1.7%) underwent angioembolisation for severe bleeding and/or haemodynamic instability, and all were from the severe LGIB group. The median length of stay was significantly longer in the severe LGIB group (5 vs. 3 days, *P* < 0.001).

Forty-eight patients (7.4%) required surgical intervention during the index admission, with a larger proportion from the severe LGIB group (10.2% vs. 5.9%, Table 5). There was no statistically significant difference in incidence of emergency surgery within 24-h between both strata. The most common operative procedures in our cohort were haemorrhoidectomy (*n* = 24/48, 50%, Table 5) and colectomy (*n* = 11/48, 22.9%). Most patients who required early surgery within 24-h had profound haemorrhoidal bleeding requiring haemorrhoidectomy and haemostasis (*n* = 8/12, 75%). One underwent a right hemicolectomy for massive bleeding from right-sided diverticula, and two underwent colostomy creation with haemostasis for large ulcerated and obstructing rectal tumours.

**Table 5:** Surgical Intervention across the cohort and within 24H of admission

Surgical intervention	n (%)
Emergency surgery during admission (total cohort)	48 (7.4)
Haemorrhoidectomy	24 (3.7)
Colectomy	11 (1.7)
Colostomy	10 (1.5)
Small bowel resection	3 (0.5)
Emergency surgery within 24H of admission	12 (1.8)
Haemorrhoidectomy, EUA and haemostasis	8 (1.2)
Colostomy	2 (0.3)
Laparotomy, enterotomy, endoscopic clipping of jejunal AVM	1 (0.2)
Right hemicolectomy	1 (0.2)

*EUA* examination under anaesthesia, *AVM* arteriovenous malformation

### Logistic regression

Table 6 shows the univariate predictors of severe LGIB. Significant predictors (*P* < 0.05) included: tachycardia (HR ≥ 100), hypotension (SBP < 90 mmHg or MAP < 65 mmHg), anaemia (Hb < 9 g/dL), low haematocrit (< 35%), metabolic acidosis (serum bicarbonate ≤ 19 mEq/dL), antiplatelet and/or anticoagulant use, active per-rectal bleeding, and acute kidney injury.

**Table 6:** Univariable and multivariable logistic regression analysis for predictive factors of severe LGIB

Variable	Univariate analysis		Multivariable analysis	
	Odds ratio (95% CI)	*P* value	Odds ratio (95% CI)	*P* value
Age			–	
> 60	1.13 (0.79–1.63)	0.5		
Gender, male	1.03 (0.75–1.43)	0.84	–	
CCM > 2	1.38 (0.97–1.95)	0.075		
CKD	1.96 (1.18–3.26)	0.01	–	
Antiplatelet/anticoagulant	1.50 (1.07–2.11)	0.02	1.93 (1.26–2.94)	0.002
Active PRB	1.80 (1.29–2.51)	0.001	2.36 (1.55–3.59)	< 0.001
HR1 ≥ 100	2.79 (1.78–4.38)	< 0.001	3.74 (2.17–6.46)	< 0.001
SBP < 90	13.91 (3.13–61.73)	0.001	15.46 (3.12–76.73)	< 0.001
MAP < 65	7.26 (2.01–26.32)	0.003	–	
Hb < 9	16.87 (10.12–28.11)	< 0.001	20.74 (11.89–36.17)	< 0.001
Hct < 35%	9.40 (6.40–13.83)	< 0.001	–	
AKI/AOCKD	2.22 (1.58–3.12)	< 0.001	–	
INR ≥ 1.5	1.97 (0.91–4.28)	0.085	–	
HCO_3_ ≤ 19	3.77 (2.00–7.10)	< 0.001	3.69 (1.65–8.22)	0.001

*CCM* Charlson Comorbidity Index, *CKD* chronic kidney disease, *PRB* PR bleeding, *HR* heart rate, *SBP* systolic blood pressure, *MAP* mean arterial pressure, *Hb* haemoglobin, *Hct* Hematocrit, *AKI* acute kidney injury, *AOCKD* acute on chronic kidney disease, *INR* international normalised ratio, *HCO*_*3*_ bicarbonate (acidosis)

**Fig. 1:**
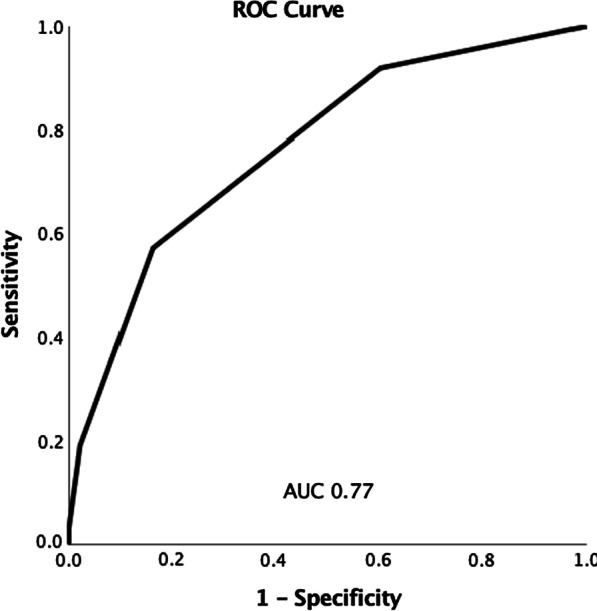
Receiver Operating Characteristics (ROC) Curve for a 6-variable prognostic model predicting severe LGIB

These variables were entered into a multivariate logistic regression model—only tachycardia, hypotension, anaemia, active per-rectal bleeding, antiplatelet and/or anticoagulant use and metabolic acidosis were statistically significant in predicting severe LGIB (Table 6). These 6 variables were used to construct a prognostic scoring model, with 1 point allocated for each risk factor (Table 7). The optimum cut-off was defined as ≥ 1 point(s), where sensitivity was 91.9%, specificity 39.8%, positive predictive value (PPV) 45.0% and negative predictive value (NPV) 90.2% in predicting severe LGIB (Table 8). The AUC (Fig. 1) of the model was 0.77 (*P* < 0.001, 95% CI 0.73–0.81).

**Table 7:** Prognostic factors for severe LGIB for inclusion in our clinical predictive model

Clinical predictive risk factor	Score (points)
Tachycardia HR ≥ 100	1
Hypotension SBP < 90 mmHg	1
Anaemia Hb < 9 g/dL	1
Active PR bleeding	1
Antiplatelet/anticoagulant use	1
Metabolic acidosis HCO_3_ ≤ 19	1

**Table 8:** Clinical predictive model for severe LGIB with sensitivity, specificity, PPV and NPV

Score	Sensitivity (%)	Specificity (%)	PPV (%)	NPV (%)
≥ 1	91.9	39.8	45	90.2
≥ 2	57	83.9	65.5	78.5
≥ 3	18.8	97.8	82.4	69.3
≥ 4	3.1	100	100	65.9
≥ 5	0.4	100	100	65.3

^*^No patient had a maximum score of 6

### Algorithm for management of LGIB

**Fig. 2:**
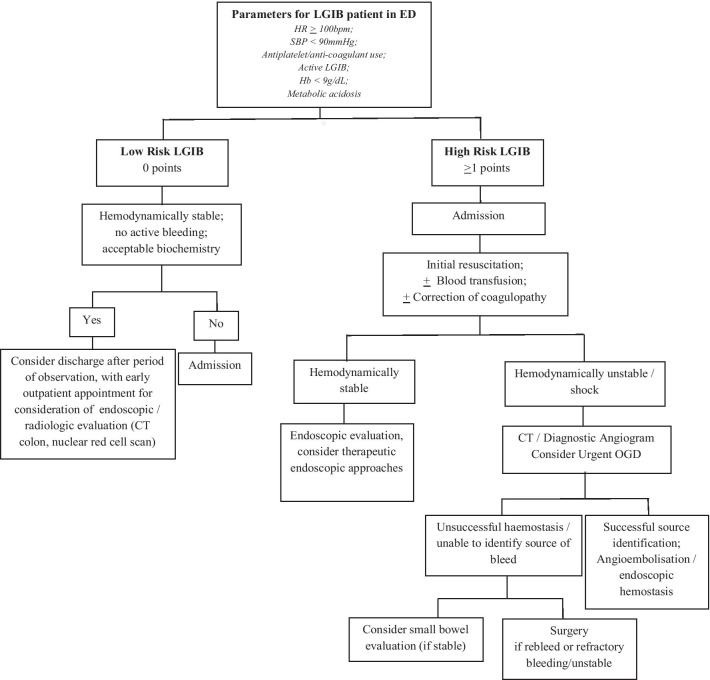
Algorithm for initial triage and management of patients presenting with LGIB

Figure 2 shows our proposed algorithm for managing patients with LGIB. Patients with 0 points derived from the multivariate model are categorised into a low-risk LGIB group. These patients can be discharged with plans for early outpatient endoscopic evaluation after a period of observation. Patients with ≥ 1 point are deemed high risk for severe LGIB and should be admitted, with hemodynamically stable patients proceeding for early endoscopic evaluation. Hemodynamically unstable patients should undergo urgent CT mesenteric angiogram (CTMA) and if indicated, angioembolisation. Once adequately resuscitated, an oesophagogastroduodenoscopy (OGD) is useful to rule out an upper gastrointestinal source; early colonoscopy can be performed in the same setting. Patients with severe LGIB that recurs or is refractory to angioembolisation and/or endoscopic intervention must be considered for colectomy.

## Discussion

Admissions for acute LGIB represent a wide spectrum of presentations from a minor bleed in hemodynamically stable patients to massive haemorrhage complicated by hypovolemic shock. Most cases of LGIB may resolve spontaneously in up to 85% of patients, allowing for potential discharge with outpatient follow up [7]. Overall prognosis is favourable, with mortality rates ranging from 2 to 10% [2, 8]. For the acute care surgeon, early dichotomisation of patients into severe versus non-severe LGIB categories may assist with timely investigations and management after initial resuscitation. In this study we have shown that our predictive model stratifies patients with severe LGIB utilising six objective variables obtained at initial presentation: active per-rectal bleed, use of antiplatelets and/or anticoagulants, tachycardia, hypotension, anaemia and/or metabolic acidosis.

Whereas multiple risk stratification systems have been validated for patients with upper gastrointestinal bleeding (UGIB), few predictive models for patients with severe LGIB currently exist. Heterogeneous resource availability and varied clinician experience worldwide has led to a lack of standardised international protocols for LGIB management. Furthermore, none have been validated in Asian populations [9]. The clinical predictive model described in the current study utilises real world and easily obtainable parameters, where the statistical likelihood of severe LGIB increases with each cumulative factor added (Table 8). Those scoring ≥ 1 point comprise a higher risk group for severe LGIB, while those scoring 0 points could potentially be managed in the outpatient setting (Fig. 2). In general, there was strong concordance of risk factors in the existing literature with the findings from our study [10].

Previous attempts have been made to risk stratify LGIB patients utilising re-bleeding, intervention rates and mortality as the end-points. In the BLEED study, re-bleeding was validated as a predictive tool for poor prognosis. Kollef et al. cited active bleed, hypotension, altered mental status and an elevated prothrombin time as predictive factors; however the tool was deemed too complex for practical use in an acute setting [11–13]. Das et al. constructed an artificial neural network (ANN) model that outperformed the BLEED criteria in predicting mortality, recurrent bleed and need for intervention. This model used non-endoscopic data made available at triage, including low haematocrit and known history of diverticular disease or arteriovenous malformation [14]. Strate et al. prospectively validated a predictive model for severe LGIB requiring 3 of 7 clinical risk factors to be satisfied—tachycardia, low systolic blood pressure, syncope, non-tender abdominal examination, per-rectal bleed in the first 4-h of medical assessment, aspirin use and more than 2 active comorbid diseases [15]. Each of these models report heterogeneous primary and secondary outcomes, limiting parallel comparisons of their performance [16]. Furthermore, some incorporated factors that may not be readily available or investigated upfront in the acute setting, such as undiagnosed diverticular disease or prothrombin time.

Thirty-day mortality was investigated as an endpoint by Sengupta et al. Advanced age, CKD, hypoalbuminemia, low haematocrit, chronic obstructive pulmonary disease, anticoagulant use, cognitive impairment and metastatic cancer were identified as independent negative prognostic factors [17]. In the current study, we found that age and CCM scores (as a surrogate marker of significant medical comorbidities) were not independently predictive of severe LGIB. Only CKD was positively correlated on multivariable analysis. Hypoalbuminemia (defined by serum albumin < 30 g/dL) was also incorporated into the HAKA score developed by Chong et al. and is generally a marker of poor nutrition and overall poor health status [18]. Its role as prognosticator for mortality has been well documented in predictive risk models for UGIB, including the Blatchford and AIMS-65 [19, 20]. However, as serum albumin is not a routine investigation for patients acutely presenting with LGIB, its role in predicting severity remains to be further elucidated.

The incidence of LGIB increases with age and associated comorbidities, presumably due to higher prevalence of diverticulosis and underlying vascular pathology [21]. The mean age in our cohort was 67, with two-thirds having 2 or more comorbidities. The higher use of anticoagulant/antiplatelet medications in this cohort (30%) may suggest why they were over-represented in the severe LGIB group. Antithrombotic therapy is associated with an increased risk of LGIB leading to bleeding from latent lesions such as colonic diverticula or arteriovenous malformations. Management of such agents should form an initial step in the treatment of LGIB. Though these medications are typically withheld following acute admission, the platelet and coagulation factor dysfunction is not easily reversed. Although warfarin reversal is well established, patients on novel anticoagulants (NOACs) remain a challenge due to the potency of these drugs and lack of a complete reversal agent [22–24]. In managing these patients, a haematologist should be consulted and fresh frozen plasma, prothrombin complex concentrate (or specific reversal agent) must be considered in cases of ongoing severe haemorrhage.

The differential diagnosis for acute LGIB can vary widely and is well published in Western literature, with the most common being diverticulosis (47.5%), colorectal polyps (20.4%) and haemorrhoids (16.9%) [25, 26]. The prevalence of colonic diverticulosis increases with age and can result in massive and recurrent bleeding between 14 and 38% of patients. In contrast to the Western population where most of the disease burden is on the left side, amongst Asians, diverticula are predominantly located in the right colon. Between 50 and 90% of all diverticular bleeding originates from the right side, which is in line with the high incidence of diverticular bleeding in our cohort, comprising almost 50% of all severe LGIB [27]. Bai et al., in a systematic analysis of 53,951 patients in the Chinese literature, reported a higher incidence of LGIB secondary to underlying colorectal malignancy (24.4%) and polyps (24.1%), with the remainder attributed to colitis (16.8%), anorectal disease (9.8%) and inflammatory bowel disease (9.5%) [28]. In the current study, we reported a higher incidence of haemorrhoidal bleeding (36.4%), of which the majority were non-severe LGIB. The higher incidence of haemorrhoidal bleeding may account for the shorter median length of stay of 3 days which in turn may result from selection bias in our local context with easier access to tertiary healthcare, as compared to other jurisdictions. Small bowel bleeding remains relatively uncommon (0.3%) but may be as high as 2–9% of LGIB in the literature, with angiodysplastic lesions being most prominent [29, 30]. It is an important differential to consider in LGIB patients with normal endoscopic findings necessitating further investigation with video capsule endoscopy or double balloon enteroscopy.

The algorithm described represents an evidence-based approach to LGIB management (Fig. 2). Colonoscopic evaluation is widely accepted as an initial modality for evaluation of LGIB. In our cohort, 55% underwent colonoscopy/flexible sigmoidoscopy, of which 39.8% were performed within 24-h. As most LGIB resolves spontaneously, colonoscopy can be performed semi-electively—by waiting for 24-h or more following admission, a patient may be optimised with blood transfusions and formal bowel preparation. Ghassemi et al. reported that urgent colonoscopy for LGIB after cleansing with bowel purge is more cost effective and associated with shorter length of stay (LOS) and higher diagnostic yield [31]. The downside, however, is that it can often be difficult to pinpoint a source after cessation of bleeding, particularly in the face of multiple co-existing pathologies such as haemorrhoids and diverticula in the elderly patient.

In our algorithm, mesenteric angiography with embolisation is reserved for hemodynamically unstable patients with refractory bleeding, and in whom there is inadequate time to await formal bowel preparation. This is supported by consensus guidelines and remains the first-line intervention for patients presenting with haemorrhagic shock [24, 32, 33]. Angiography can detect bleeding rates down to 0.5–1.0 ml/min, and location of bleeding of angiography before successful embolisation is associated with a reduced risk of re-bleeding [34]. Where amenable, super-selective angioembolisation has become more widely advocated for its greater safety profile, with lower rates of ischemic complications and bowel infarction. However, this is a technically demanding procedure that requires specialist expertise, which may not be available in all institutions. An urgent OGD should also be considered to rule out a brisk bleeding source proximal to the ligament of Treitz.

Patients requiring urgent colectomy for LGIB have decreased significantly over the years due to advances in endoscopic haemostasis and angio-embolisation techniques. Surgery is undertaken in our institution for patients with recurrent or refractory bleeding, unsuccessful endoscopic haemostasis or obscure LGIB without an identifiable source and those who are unstable despite resuscitation and medical optimisation. None of our patients required a blind subtotal colectomy, which may be performed in cases where massive LGIB is attributed to an unidentifiable colonic source, for example in a patient with pan-diverticulosis. However, this procedure is historically associated with high morbidity and mortality rates and generally serves as a last resort [35, 36].

There are limitations to our retrospective analysis. The cohort is derived from a single tertiary institution involving patients admitted to surgical services. Those discharged directly from ED were not captured and may have contributed to a selection bias. The counter argument is that patients deemed fit for discharge from ED were likely at inherent “low risk” for severe LGIB and may not have had a significant impact on our predictive model. The model was constructed from a derivative cohort and needs to be externally validated in a prospective cohort, limiting the generalisability of our findings. Our study utilised a cut-off of SBP 90 mmHg to define hypotension. In reality, baseline population SBP increases with advancing age [37]. The concept of “relative hypotension” is patient specific, and should be considered when triaging each patient with LGIB. Finally, the predictive factors studied are non-exhaustive, and confounders of prognostic significance may exist, which have yet to be identified.

Overall, our study contributes to the existing literature by evaluating real world and easily accessible clinical and pre-endoscopic factors for risk-stratification of patients with LGIB. To our knowledge, it remains the first Asian study to do so. The ROC curve reflected high predictive accuracy and in those patients with a threshold of ≥ 1 point(s), the model showed high sensitivity and NPV. Hence, the model was strongest for “ruling out” a severe bleed, which can guide potential discharge of a low-risk patient. The proposed model can be easily implemented to aid in clinical decision making, allowing for early identification of severe LGIB patients who require aggressive resuscitation, admission to a monitored bed and consideration of endoscopic or surgical intervention. Besides its use in initial triage, the algorithm is also relevant when encountering changes in clinical trajectory of patients with LGIB. For example, if a stable patient planned for early inpatient endoscopy develops massive per-rectal bleeding with haemodynamic compromise, they should be moved from the original pathway to the “haemodynamically unstable” arm, and proceed with an urgent CT angiogram instead.

## Conclusion

Timely diagnosis and management of severe LGIB remains a challenge. The acute care surgeon needs to recognise this clinical entity early and determine the need for urgent endoscopic evaluation and/or angio-embolisation and surgery. The clinical predictive model for severe LGIB described utilises objective clinical parameters routinely obtained at initial evaluation. Further studies are needed to externally validate this model in a prospective cohort.

## Data Availability

The datasets during and/or analysed during the current study available from the corresponding author on reasonable request.

## Abbreviations

LGIB: Lower gastrointestinal bleed
AUC: Area under curve
ED: Emergency Department
UGIB: Upper gastrointestinal bleed
OR: Odds ratio
PPV: Positive predictive value
NPV: Negative predictive value
ROC: Receiver operating characteristics
CCM: Charlson Comorbidity Index
CKD: Chronic kidney disease
NSAID: Non-steroidal anti-inflammatory drug
HR: Heart rate
SBP: Systolic blood pressure
DBP: Diastolic blood pressure
MAP: Mean arterial pressure
HB: Haemoglobin
HCT: Haematocrit
AKI: Acute kidney injury
INR: International normalised ratio
HCO_3_: Serum bicarbonate
SRUS: Solitary rectal ulcer syndrome
AVM: Arteriovenous malformation
PCT: Packed cell transfusion
OGD: Oesophago Gastro Duodenoscopy
IQR: Interquartile range
ICU: Intensive care unit
EUA: Examination under anaesthesia
NOAC: Novel Oral Anticoagulant
LOS: Length of stay
